# Supplementary material for: Soil carbon sequestration accelerated by restoration of grassland biodiversity
Source: Nat Commun. 2019 Feb 12;10:718. doi: 10.1038/s41467-019-08636-w (PMC6372642; doi:10.1038/s41467-019-08636-w)
Supplement: Supplementary file 1 — Supplementary Information [file 41467_2019_8636_MOESM1_ESM.pdf]

Supplementary Information for

Soil carbon sequestration accelerated by restoration of grassland biodiversity

Yi Yang[1], David Tilman[1,2*], George Furey[1] and Clarence Lehman[1]

1 Department of Ecology, Evolution, and Behavior, University of Minnesota, St. Paul, MN 55108, USA.

2 Bren School of Environmental Science and Management, University of California, Santa Barbara, 93106, USA.

*To whom correspondence should be addressed. E-mail: tilman@umn.edu

This PDF file includes:

Supplementary Tables 1 to 13

Supplementary Figures 1 to 4

References

Supplementary Table 1. Decadal soil C storage rates, as quantified by ΔC/Δt (units of Mg-C ha^{-1} yr^{-1}), with standard errors (s.e.).

**Number of species**	**Decade**	**Sample size**	**Soil C storage rate (mean (s.e.))**	
			**0-20 cm**	**0-60 cm**
1	yrs 1-13	30	-0.01 (0.05)	0.08 (0.07)
1	yrs 13-22	30	0.28 (0.07)	0.42 (0.10)
2	yrs 1-13	28	0.09 (0.05)	0.27 (0.08)
2	yrs 13-22	28	0.30 (0.10)	0.47 (0.14)
4	yrs 1-13	29	0.16 (0.05)	0.38 (0.09)
4	yrs 13-22	29	0.33 (0.06)	0.60 (0.12)
8	yrs 1-13	30	0.11 (0.06)	0.29 (0.10)
8	yrs 13-22	30	0.46 (0.07)	0.73 (0.11)
16	yrs 1-13	35	0.18 (0.05)	0.54 (0.08)
16	yrs 13-22	35	0.51 (0.07)	0.71 (0.11)

Supplementary Table 2. Mean soil C concentration (%) measured in different years for different species diversities, with standard errors (s.e.). The upper 20 cm was measured 5 times (in 1994, 2000, 2004, 2006, 2015), and depths below 20 cm sampled 3 times (in 1994, 2006, 2015).

**Number of Species**	**Year**	**Sample size**	**Soil C concentration (%) (mean (s.e.))**		
			**0-20 cm**	**20-40 cm**	**40-60 cm**
1	1994	30	0.49 (0.02)	0.31 (0.02)	0.23 (0.01)
1	2000	30	0.44 (0.02)		
1	2004	30	0.45 (0.02)		
1	2006	30	0.48 (0.02)	0.34 (0.02)	0.24 (0.01)
1	2015	30	0.57 (0.03)	0.36 (0.02)	0.26 (0.02)
2	1994	28	0.50 (0.02)	0.34 (0.02)	0.25 (0.01)
2	2000	28	0.47 (0.02)		
2	2004	28	0.50 (0.02)		
2	2006	28	0.54 (0.03)	0.39 (0.02)	0.28 (0.01)
2	2015	28	0.64 (0.04)	0.43 (0.02)	0.29 (0.01)
4	1994	29	0.46 (0.03)	0.32 (0.02)	0.22 (0.02)
4	2000	29	0.48 (0.02)		
4	2004	29	0.50 (0.02)		
4	2006	29	0.54 (0.03)	0.35 (0.02)	0.28 (0.02)
4	2015	29	0.64 (0.04)	0.43 (0.03)	0.29 (0.02)
8	1994	30	0.50 (0.03)	0.34 (0.02)	0.23 (0.02)
8	2000	30	0.51 (0.03)		
8	2004	30	0.54 (0.02)		
8	2006	30	0.55 (0.02)	0.38 (0.02)	0.28 (0.02)
8	2015	30	0.70 (0.03)	0.44 (0.02)	0.30 (0.02)
16	1994	35	0.52 (0.03)	0.31 (0.02)	0.23 (0.02)
16	2000	35	0.52 (0.03)		
16	2004	35	0.56 (0.03)		
16	2006	35	0.60 (0.03)	0.40 (0.03)	0.30 (0.02)
16	2015	35	0.76 (0.04)	0.45 (0.03)	0.31 (0.02)

Supplementary Table 3. Mean soil C stocks (Mg-C ha^{-1}) in 1994, 2006, and 2015 for different species diversities (see Methods), with standard errors (s.e.).

**Number of species**	**Year**	**Sample size**	**Soil C stocks (mean (s.e.))**	
			**0-20 cm**	**0-60 cm**
1	1994	30	14.7 (0.7)	31.5 (1.4)
1	2006	30	14.5 (0.7)	32.6 (1.5)
1	2015	30	17.1 (0.8)	36.3 (1.8)
2	1994	28	15.1 (0.6)	33.0 (1.3)
2	2006	28	16.3 (0.8)	36.5 (1.5)
2	2015	28	19.0 (1.1)	40.7 (1.8)
4	1994	29	14.0 (0.8)	30.5 (1.7)
4	2006	29	16.1 (0.9)	35.4 (1.8)
4	2015	29	19.1 (1.0)	40.9 (2.3)
8	1994	30	15.2 (0.9)	32.0 (1.8)
8	2006	30	16.6 (0.7)	35.8 (1.5)
8	2015	30	20.7 (0.8)	42.4 (1.9)
16	1994	35	15.6 (0.9)	31.8 (2)
16	2006	35	18.0 (1.0)	38.7 (2.2)
16	2015	35	22.5 (1.2)	45.2 (2.6)

Supplementary Table 4. Mean soil C storage rates (C: Mg ha^{-1} yr^{-1}) and growth rates (% yr^{-1}) throughout the entire 22 years (0-60 cm), with standard errors (s.e.).

**Number of species**	**Sample size**	**Annual change (mean (s.e.))**	
		**Storage rates** $\Delta C/\Delta t$	**Growth rates** $\ln(C_{t+\Delta t}/C_t)/\Delta t$
1	30	0.22 (0.04)	0.6 (0.1)
2	28	0.35 (0.06)	1.0 (0.2)
4	29	0.47 (0.06)	1.3 (0.2)
8	30	0.47 (0.05)	1.3 (0.1)
16	35	0.61 (0.05)	1.6 (0.1)

Supplementary Table 5. Linear mixed model (Restricted Maximum Likelihood) for log of soil C concentration (as %) in upper 20 cm of soil as a function of T (number of years since 1993), T^2, S (number of planted species), and T*S interaction, with plot as random effect. N=760

Fixed Effect Tests

**Source**	**DF**	**DFDen**	**F Ratio**	**Prob > F**
T^2	1	605	60.0	<.0001
T	1	605	2.1	0.1508
S	1	150	9.5	0.0025
S*T	1	605	24.5	<.0001

Parameter Estimates

**Variable**	**Coefficient**	**Std Error**	**t Ratio**	**p**
Intercept	-0.8133	0.0300	-27.12	<.0001
T^2	0.0008	0.0001	7.74	<.0001
T	-0.0036	0.0025	-1.44	0.1508
S	0.0097	0.0032	3.08	0.0025
(T-10.8)*(S-6.6)	0.0007	0.0001	4.95	<.0001

Supplementary Table 6. Multiple regression analysis for decadal soil C sequestration rates (as Mg-C ha^{-1} yr^{-1}) in upper 60 cm of soil as a function of S (number of planted species) and decade (as a categorical variable).

Parameter estimate				
**Term**	**Estimate**	**Std Error**	**t Ratio**	**p**
Intercept	0.3180	0.0486	6.55	<.0001
decade[yrs 1-13]	-0.1357	0.0317	-4.29	<.0001
S	0.0208	0.0056	3.72	0.0002
decade[yrs 1-13]*(S-6.6)	0.0022	0.0056	0.39	0.6939
Summary of fit				
R Squared	0.10			
R Squared Adj	0.09			
Root Mean Square Error	0.55			
Mean of Response	0.45			
Observations (or Sum Wgts)	304			
Analysis of variance				
**Source**	**DF**	**Sum of Squares**	**Mean Square**	**F Ratio**
Model	3	9.86	3.29	10.78
Error	300	91.42	0.30	
C. Total	303	101.28		<.0001

Supplementary Table 7. Multiple regression analysis for decadal soil C sequestration rates (as Mg-C ha^{-1} yr^{-1}) in upper 20 cm of soil as a function of S (number of planted species) and decade (as a categorical variable).

Parameter estimate				
**Term**	**Estimate**	**Std Error**	**t Ratio**	**p**
Intercept	0.1649	0.0311	5.3	<.0001
decade[yrs 1-13]	-0.1364	0.0203	-6.73	<.0001
S	0.0122	0.0036	3.42	0.0007
decade[yrs 1-13]*(S-6.6)	-0.0033	0.0036	-0.93	0.3552
Summary of fit				
R Squared	0.16			
R Squared Adj	0.15			
Root Mean Square Error	0.35			
Mean of Response	0.25			
Observations (or Sum Wgts)	304			
Analysis of variance				
**Source**	**DF**	**Sum of Squares**	**Mean Square**	**F Ratio**
Model	3	7.23	2.41	19.26
Error	300	37.52	1.13	
C. Total	303	44.75		<.0001

Supplementary Table 8. Linear mixed model (Restricted Maximum Likelihood) for root C content (as Mg-C ha^{-1}) in upper 30 cm of soil as a function of T (number of years since 1993), S (number of planted species), and T*S interaction, with plot as random effect. N=1929

Fixed Effect Tests				
**Source**	**DF**	**DFDen**	**F Ratio**	**Prob > F**
T	1	1768	376.54	<.0001
S	1	161	108.32	<.0001
T*S	1	1766	55.22	<.0001
Parameter Estimates				
**Variable**	**Coefficient**	**Std Error**	**t Ratio**	**p**
Intercept	1.1903	0.1226	9.71	<.0001
T	0.0677	0.0035	19.4	<.0001
S	0.1442	0.0139	10.41	<.0001
(T-11.2)*(S-6.3)	0.0046	0.0006	7.43	<.0001

Supplementary Table 9. Linear mixed model (Restricted Maximum Likelihood) for root (0-30 cm) to shoot ratio as a function of T (number of years since 1993), S (number of planted species), and presence [1] or absence [0] of species in 4 functional groups, with plot as random effect. N=1820

Fixed Effect Tests				
**Source**	**DF**	**DFDen**	**F Ratio**	**Prob > F**
T	1	1660	116.82	<.0001
S	1	162	3.33	0.0701
C3	1	162	4.84	0.0293
C4	1	162	166.15	<.0001
Forb	1	165	14.15	0.0002
Legume	1	163	97.00	<0.0001
Parameter Estimates				
**Variable**	**Coefficient**	**Std Error**	**t Ratio**	**p**
Intercept	-196.2828	18.5402	-10.59	<.0001
T	0.1000	0.0093	10.81	<.0001
S	-0.0493	0.0270	-1.82	0.0701
C3[0]	-0.2295	0.1043	-2.2	0.0293
C4[0]	-1.2521	0.0971	-12.89	<.0001
Forb[0]	0.3952	0.1051	3.76	0.0002
Legume[0]	0.9995	0.1015	9.85	<.0001

Supplementary Table 10. Linear mixed model (Restricted Maximum Likelihood) for soil C concentration (as %) in upper 20 cm of soil as a function of P (aboveground productivity in Mg ha^{-1}), R (root biomass in Mg ha^{-1}), and T (number of years since 1993), with plot as random effect. N=608

Fixed Effect Tests				
**Source**	**DF**	**DFDen**	**F Ratio**	**Prob > F**
P	1	586	8.72	0.0033
R	1	597	12.78	0.0004
T	1	442	393.62	<.0001
Parameter Estimates				
**Variable**	**Coefficient**	**Std Error**	**t Ratio**	**p**
Intercept	0.3114	0.0190	16.36	<.0001
P	0.0157	0.0053	2.95	0.0033
R	0.0061	0.0017	3.57	0.0004
T	0.0120	0.0006	19.84	<.0001

Supplementary Table 11. Multiple regression analysis for soil C storage rates over the 22 years (1994-2015) in upper 60 cm of soil as a function of mean species abundance (g m^{-1}) of the previous 10 years (2006-2015, with first sample done in 2001).

Parameter estimate				
**Term**	**Estimate**	**Std Error**	**t Ratio**	**p**
Intercept	0.0878	0.0539	1.63	0.1055
Achillea millefolium(lanulosa)	-0.0024	0.0039	-0.61	0.5442
Amorpha canescens	-0.0006	0.0020	-0.29	0.7686
Andropogon gerardi	0.0023	0.0005	4.91	<.0001***
Asclepias tuberosa	-0.0147	0.0072	-2.03	0.0445
Elymus canadensis	0.0067	0.0584	0.11	0.9088
Koeleria cristata	0.0030	0.0016	1.91	0.058
Lespedeza capitata	0.0021	0.0007	2.93	0.004***
Liatris aspera	0.0009	0.0006	1.34	0.1827
Lupinus perennis	0.0014	0.0014	1	0.3176
Monarda fistulosa	0.0029	0.0022	1.31	0.1928
Panicum virgatum	0.0019	0.0009	2.12	0.0357*
Petalostemum purpureum	0.0015	0.0006	2.64	0.0092**
Petalostemum villosum	0.0007	0.0010	0.67	0.5026
Poa pratensis	0.0049	0.0023	2.17	0.0318*
Schizachyrium scoparium	0.0012	0.0007	1.83	0.0687
Solidago rigida	0.0004	0.0009	0.43	0.6696
Sorghastrum nutans	0.0022	0.0008	2.64	0.0092**
Summary of fit				
R Squared	0.503			
R Squared Adj	0.439			
Root Mean Square Error	0.237			
Mean of Response	0.430			
Observations (or Sum Wgts)	152			
Analysis of variance				
**Source**	**DF**	**Sum of Squares**	**Mean Square**	**F Ratio**
Model	17	7.63	0.45	7.98
Error	134	7.54	0.06	
C. Total	151	15.16		<.0001

Supplementary Table 12: The pool of native perennial species from which each diversity combination was selected.

Species	Functional type
Lupinis perennis	Legume
Andropogon gerardi	C4 grass
Schizachyrium scoparium	C4 grass
Sorghastrum nutans	C4 grass
Solidago rigida	Forb
Amorpha canescens	Legume (woody shrub)
Lespedeza capitata	Legume
Poa pratensis	C3 grass
Petalostemum purpureum	Legume
Monarda fistulosa	Forb
Achillea millefolium	Forb
Panicum virgatum	C4 grass
Liatris aspera	Forb
Quercus macrocarpa	Woody
Koeleria cristata	C3 grass
Quercus elipsoidalis	Woody
Elymus canadensis	C3 grass
Agropyron smithii	C3 grass

Supplementary Table 13. Mean bulk density, measured in 2018, at 0-20 cm, 20-40 cm, and 40-60 cm for each diversity treatment. Letters beneath means and standard errors indicate statistics differences based on Tukey's Honest Significant Difference (HSD) test: those sharing the same letter are not statistically different (P<0.05).

**Number of species**	**Sample size**	**Bulk density (mean (s.d.))**		
		**0-20 cm**	**20-40 cm**	**40-60 cm**
1	26	1.47 (.02)<br>d	1.68 (.01)<br>abc	1.73 (.02)<br>abc
2	16	1.43 (.01)<br>de	1.64 (.03)<br>bc	1.76 (.03)<br>a
4	15	1.42 (.02)<br>de	1.66 (.02)<br>abc	1.72 (.02)<br>abc
8	15	1.36 (.02)<br>e	1.64 (.02)<br>bc	1.70 (.02)<br>abc
16	15	1.38 (.02)<br>e	1.63 (.02)<br>c	1.69 (.02)<br>abc

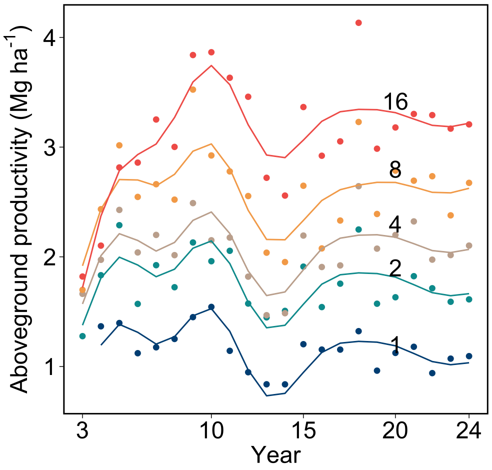

Supplementary Fig. 1 | Change in aboveground productivity over time. Dots are means of a given year, and lines are fitted using a generalized additive model (GAM) with a smoother for each level of plant diversity (indicated by the numbers).

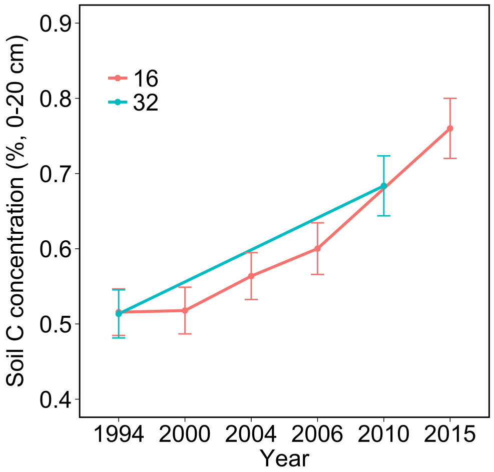

Supplementary Fig. 2 | Plots of 32 native prairie species (blue) of a nearby experiment, never weeded, had similar rates of soil C sequestration as did the 16-pecies plots in our experiment (red). Dots are means with standard errors. The 32-species plots were sampled in 1994 and 2010.

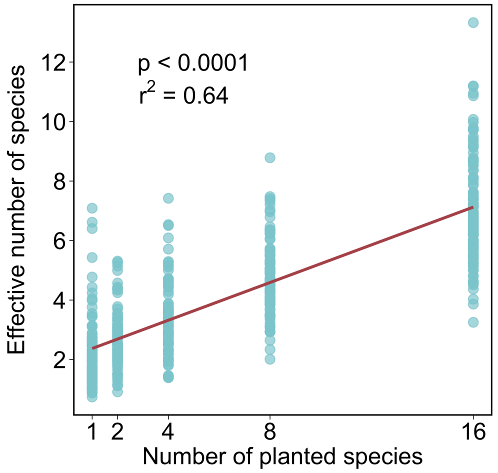

Supplementary Fig. 3 | Correlation between current species richness from 2013 to 2015, as estimated by the effective number of species, and the number of planted species.

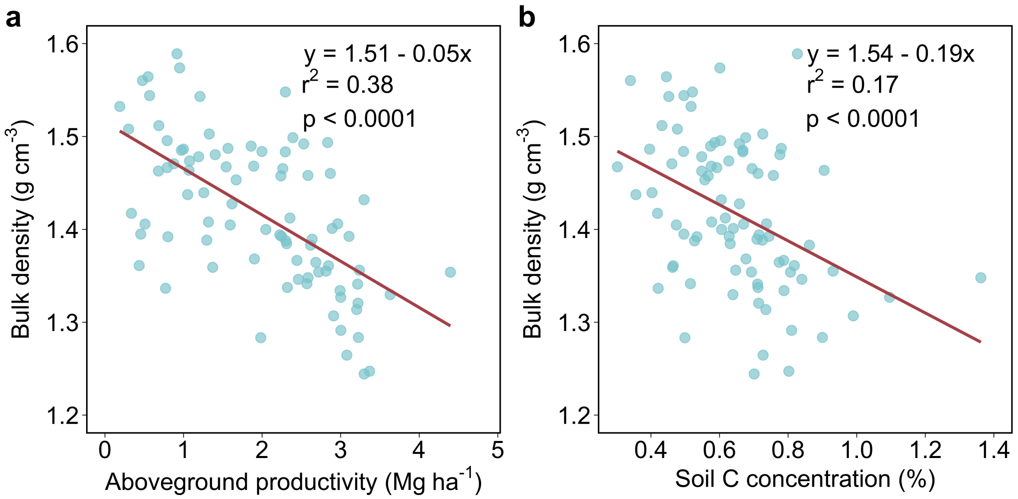

Supplementary Fig. 4 | Linear regression analysis of bulk density for each plot (year 2018) as a function of aboveground productivity (a, year 2017) and mean soil C concentration (b, year 2015). Aboveground productivity yields a better r^2 (0.38), and the intercept (1.51) can be interpreted as the bulk density when productivity is 0 (at the time of planting in 1994).
